# Identification of Patients With Congestive Heart Failure From the Electronic Health Records of Two Hospitals: Retrospective Study

**DOI:** 10.2196/64113

**Published:** 2025-04-10

**Authors:** Daniel Sumsion, Elijah Davis, Marta Fernandes, Ruoqi Wei, Rebecca Milde, Jet Malou Veltink, Wan-Yee Kong, Yiwen Xiong, Samvrit Rao, Tara Westover, Lydia Petersen, Niels Turley, Arjun Singh, Stephanie Buss, Shibani Mukerji, Sahar Zafar, Sudeshna Das, Valdery Moura Junior, Manohar Ghanta, Aditya Gupta, Jennifer Kim, Katie Stone, Emmanuel Mignot, Dennis Hwang, Lynn Marie Trotti, Gari D Clifford, Umakanth Katwa, Robert Thomas, M Brandon Westover, Haoqi Sun

**Affiliations:** 1 Department of Neurology Beth Israel Deaconess Medical Center Boston, MA United States; 2 Brigham Young University Provo, UT United States; 3 Department of Neurology Massachusetts General Hospital Boston, MA United States; 4 Department of Health Outcomes and Biomedical Informatics College of Medicine University of Florida Gainesville, FL United States; 5 Northeastern University Boston, MA United States; 6 Utrecht University Utrecht University Utrecht The Netherlands; 7 Boston College Boston, MA United States; 8 Yale School of Medicine New Haven, CT United States; 9 California Pacific Medical Center Research Institute San Francisco, CA United States; 10 Department of Epidemiology and Biostatistics University of California San Francisco, CA United States; 11 Stanford University Stanford, CA United States; 12 Kaiser Permanente Fontana, CA United States; 13 Emory University Atlanta, GA United States; 14 Georgia Institute of Technology Atlanta, GA United States; 15 Boston Children's Hospital Boston, MA United States; 16 Division of Pulmonary, Critical Care and Sleep Medicine Department of Medicine Beth Israel Deaconess Medical Center Boston United States

**Keywords:** electronic health record, machine learning, artificial intelligence, phenotype, congestive heart failure, medication, claims database, International Classification of Diseases, effectiveness, natural language processing, model performance, logistic regression, validity

## Abstract

**Background:**

Congestive heart failure (CHF) is a common cause of hospital admissions. Medical records contain valuable information about CHF, but manual chart review is time-consuming. Claims databases (using *International Classification of Diseases* [*ICD*] codes) provide a scalable alternative but are less accurate. Automated analysis of medical records through natural language processing (NLP) enables more efficient adjudication but has not yet been validated across multiple sites.

**Objective:**

We seek to accurately classify the diagnosis of CHF based on structured and unstructured data from each patient, including medications, *ICD* codes, and information extracted through NLP of notes left by providers, by comparing the effectiveness of several machine learning models.

**Methods:**

We developed an NLP model to identify CHF from medical records using electronic health records (EHRs) from two hospitals (Mass General Hospital and Beth Israel Deaconess Medical Center; from 2010 to 2023), with 2800 clinical visit notes from 1821 patients. We trained and compared the performance of logistic regression, random forests, and RoBERTa models. We measured model performance using area under the receiver operating characteristic curve (AUROC) and area under the precision-recall curve (AUPRC). These models were also externally validated by training the data on one hospital sample and testing on the other, and an overall estimated error was calculated using a completely random sample from both hospitals.

**Results:**

The average age of the patients was 66.7 (SD 17.2) years; 978 (54.3%) out of 1821 patients were female. The logistic regression model achieved the best performance using a combination of *ICD* codes, medications, and notes, with an AUROC of 0.968 (95% CI 0.940-0.982) and an AUPRC of 0.921 (95% CI 0.835-0.969). The models that only used *ICD* codes or medications had lower performance. The estimated overall error rate in a random EHR sample was 1.6%. The model also showed high external validity from training on Mass General Hospital data and testing on Beth Israel Deaconess Medical Center data (AUROC 0.927, 95% CI 0.908-0.944) and vice versa (AUROC 0.968, 95% CI 0.957-0.976).

**Conclusions:**

The proposed EHR-based phenotyping model for CHF achieved excellent performance, external validity, and generalization across two institutions. The model enables multiple downstream uses, paving the way for large-scale studies of CHF treatment effectiveness, comorbidities, outcomes, and mechanisms.

## Introduction

Congestive heart failure (CHF) and all types of heart disease are the primary causes of hospital admissions and the leading cause of death in the United States [1-3]. The prevalence of CHF in the United States is estimated to be 7% for men and 4.2% for women [4]. As a result, it is important to diagnose CHF early and accurately, which is difficult given the diversity of CHF and the lack of definitive, universally applicable diagnostic tests [5]. This underscores the need for research on improving diagnosis and predicting patient outcomes. The heterogeneity of CHF makes it hard to design clinical trials that can accurately and uniformly diagnose CHF [6]. In this context, accurate classification is crucial for the reliability of epidemiological and outcomes research. Traditionally, the identification of CHF is a labor-intensive process. Local sites identify potential cases and forward medical records to a Central Clinical Events Committee, comprised of physician experts, who manually review each case against established standards [7-9]. While this method is considered the gold standard, it is impractical for large observational studies, where *International Classification of Diseases* (*ICD*) codes are typically used. However, sole reliance on *ICD* codes is problematic due to their imprecision; it is estimated that approximately 40% of hospitalizations labeled with primary *ICD* codes for CHF do not hold up upon detailed medical record review [9]. Consequently, there is a growing interest in automated methods for adjudicating CHF hospitalizations from medical records that could streamline the process; reduce reliance on physician-based Central Clinical Events Committee reviews; foster the development of large, cost-effective clinical trials; and help gather more accurate data for research [9].

In this context, electronic health records (EHRs) have become invaluable. Encompassing millions of patient profiles, these records are stored across various hospitals and institutions as structured and unstructured data. Much of the data exists as unstructured natural language in physicians’ notes, documenting symptoms, conditions, and diagnoses. Natural language processing (NLP) approaches present an opportunity to harness this rich source of data, enabling high-throughput identification and phenotyping of patients directly from hospital databases [10-13]. Some applications of these models in inpatient fall detection [14], emergency triage notes [15], and inpatient fall risk assessment [16] have already achieved a high level of effectiveness using a variety of modeling methods, including large language models such as Bidirectional Encoder Representations from Transformers, showing the need for further development in other medical applications.

Here, we address these gaps by introducing a comprehensive approach to EHR-based phenotyping of CHF within a large multihospital cohort, based on clinical notes, *ICD* codes, and medications, contributing to the growing body of knowledge in automated medical record review and phenotyping of patients with CHF, as well as ensuring reproducible and reliable clinical use [17,18].

## Methods

### Cohort

We retrospectively obtained EHR data from two sites: Beth Israel Deaconess Medical Center (BIDMC) and Massachusetts General Hospital (MGH), from 2010 to 2023. The EHR data contain clinical notes written by the clinician, *ICD* diagnosis codes (*ICD-9* and *ICD-10*) assigned during encounters, and prescribed medications. To enable initial cohort selection and maximize the chance of including patients with CHF from this large pool of inpatient and outpatient encounters, we divided patients into groups based on the presence of prespecified *ICD* codes (“*ICD*+” or “*ICD*–”) related to CHF and prespecified medications (“MED+” or “MED–”), creating four groups: “*ICD*+/MED+,” “*ICD*–/MED+,” “*ICD*+/MED–,” and “*ICD*–/MED–.” We then randomly sampled a total of 1400 notes with keywords (such as heart, CHF, etc) from the four groups at each site to undergo manual annotation (described below) to provide labels for model training. This sample size was selected to enable appropriate training and to not overwhelm the available computing power.

### Ethical Considerations

The retrospective data were obtained under Beth Israel Deaconess Medical Center's institutional review board that waived the need for informed consent (BIDMC IRB# 2022P000481).

### CHF-Related Keywords, ICD Codes, and Medications for Initial Cohort Selection

*ICD* codes and medications related to CHF were specified as a part of the model input. *ICD* codes included 428 (*ICD-9*) for “Congestive Heart Failure, unspecified” and I50 (*ICD-10*) for “Heart Failure.” The medications are shown in Table 1. We also specified CHF-related keywords to be highlighted during manual note review, to facilitate the annotation of ground-truth CHF status and to include them as classifying features (see below). The keywords were determined using two approaches. The first was the knowledge-driven approach: we obtained as many keywords as possible from medical doctors and by manually reviewing published papers. This was done by simply reading papers and selecting words that appeared frequently and were related to CHF and then presenting them to physicians to check for accuracy (see references for links to papers). The second was the data-driven approach: we listed all unique words (1-gram), consecutive two-word sequences (2-gram), and consecutive three-word sequences (3-gram) in all notes. Then, using the associated, CHF-related *ICD* codes as a rough approximation of ground-truth CHF status, for each n-gram, we conducted a Mann-Whitney *U* test to compare the median number of occurrences in the groups with and without CHF. Words that occurred with significantly different frequencies between groups were included in the keyword list. We further expanded the set of potentially classifying features by augmenting keywords and phrases with their negations.

**Table 1:** Medications related to congestive heart failure.

Medication generic name	Medication description
Lisinopril	Angiotensin-converting enzyme inhibitor
Furosemide	Diuretic
Bumetanide	Diuretic
Propranolol	Beta-blocker
Amlodipine	Calcium channel blocker
Hydrochlorothiazide	Diuretic
Omeprazole	Proton pump inhibitor
Isosorbide mononitrate	Vasodilator
Enalapril	Angiotensin-converting enzyme inhibitor

### Annotation of the Ground-Truth CHF Status

Ground-truth CHF status was based on having human annotators read through clinical notes and make a yes-or-no decision for each note as to whether the note author affirmed a diagnosis of CHF in the note. To ease annotation, we displayed each clinical note with keywords highlighted so that the annotator could more quickly determine CHF status by reading the note. Notes were randomly assigned to 5 independent annotators. We followed a standard operating procedure to ensure consistency between raters. Notes were randomly split between 5 annotators without overlap. Patients who were said to have CHF or a “history of CHF” were labeled as “positive” cases. All others were labeled as “negative” cases. This included patients noted as being “unlikely [to have] CHF,” patients with a family history but no personal diagnosis of CHF, and patients with no indication of CHF given in the note.

### Feature Matrix Construction

Features constructed to train the NLP model included three subsets: features based on keywords and/or phrases in the notes; features based on *ICD* codes; and features based on medications. To construct features based on notes, we applied stemming and lemmatization. The number of occurrences for each keyword or phrase in the note was then defined as the feature value. Features based on *ICD* codes and medications were identified as the number of occurrences in the medical record dated within 18 months before or after the note’s date.

### Model Development: Logistic Regression, Random Forests, and RoBERTa

We developed models based on different subsets of information and with different model architectures. The first model was a logistic regression model. The training was done using nested, 5-fold cross-validation with an elastic-net penalty, using Bayesian optimization to fine-tune the hyperparameters (penalty strength and L1 ratio) to achieve the maximum average area under the receiver operating characteristic curve (AUROC) across all inner validation sets. For each outer fold, a model was fitted with the best hyperparameters, and a receiver operating characteristic curve was obtained using the outer testing set. Using the Youden index, we determined a probability cutoff value for binary classification in each outer fold. The final performance metric was calculated as the average of the 5 AUROCs from the outer folds. We also trained a random forests classifier using the same approach with nested, 5-fold cross-validation, using Bayesian optimization to fine-tune the hyperparameters (minimum leaves per sample, number of estimators, maximum depth, and cost-complexity pruning coefficient) to incorporate ensemble methods because of their robustness to reduce bias [19]. We finally trained a deep learning model based on RoBERTa followed by logistic regression. We used the embeddings from RoBERTa as our feature matrix with no additional pretraining, as previous studies have shown that RoBERTa has already been optimized [20]. We did this in three different ways. First, we split the note into sections of up to 512 tokens with each section having a one-sentence overlap. We then took the vector representations of these segments and took the elementwise average to get a feature matrix. Second, we took 250 tokens forward and backward from the keywords “CHF” or “congestive heart failure” and found the embeddings from these sections. Third, if multiple keywords were found, then the elementwise average was taken. If no keywords were found, then all columns were set to zero.

For model type, we tested logistic regression, random forests, and RoBERTa, spanning linear and nonlinear models, as well as simple statistical and deep learning models. While large language models are powerful tools, they are not always necessary, nor are they always the best approach. Simpler approaches often demonstrate that excellent results can be achieved without the additional complexity.

### Feature Importance

**Figure 1:**
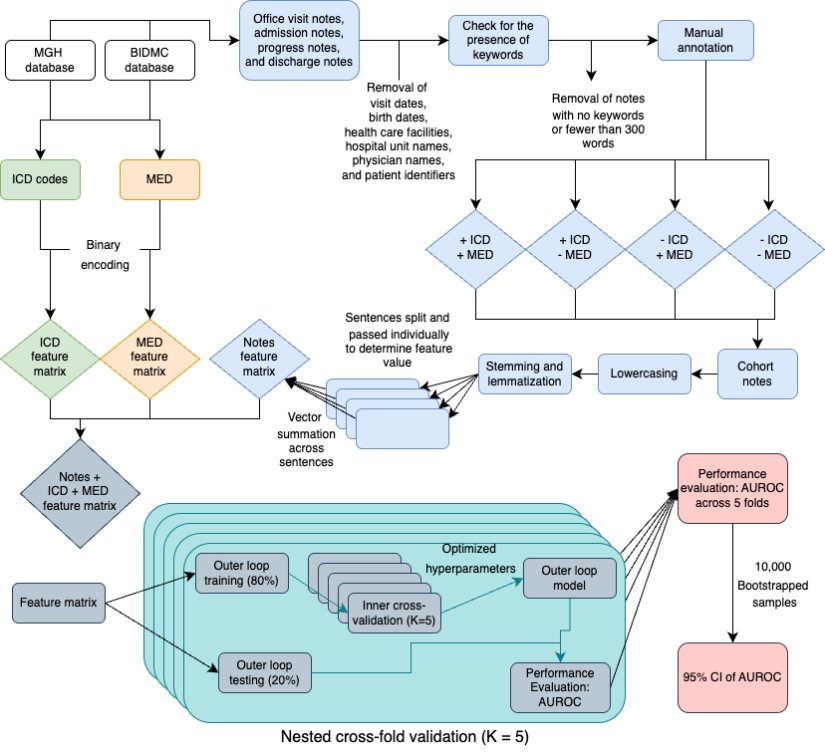
Flowchart demonstrating the data collection, methods of feature matrix construction, and model usage. AUROC: area under the receiver operating characteristic curve; BIDMC: Beth Israel Deaconess Medical Center; ICD: International Classification of Diseases; MED: medications; MGH: Mass General Hospital.

All models used to derive feature importance were trained using median hyperparameters across the 5 outer cross-validation folds and trained on the entire dataset. For logistic regression, feature importance was given by model coefficients. For random forests, feature importance was measured by the total reduction of the Gini impurity associated with that feature, normalized to sum up to 1 across all features. We did not investigate feature importance from RoBERTa since it did not achieve better performance than logistic regression or random forests (see the *Results* section). Figure 1 summaries the methods into a visual aid.

### Statistical Analysis

Statistical significance was set at *P*<.05. Further, 95% CIs were determined using 10,000 rounds of bootstrapping. All analyses were performed using Python (version 3.10.13; Python Software Foundation).

## Results

### Cohort Characteristics

The final cohort included 1821 patients with 2800 notes (Table 2). The average age was 66.7 (SD 17.2) years; 978 (54.3%) out of 1821 patients were female. The racial and ethnic composition of the sample was 3.8% (69/1821) Black or African American, 6.3% (115/1821) Hispanic, 1.9% (35/1821) Asian, and 80.7% (1471/1821) White. The three most common medications used were omeprazole, furosemide, and lisinopril. The most common *ICD* code stem was I50. In terms of institutions, of the 2800 notes, 1440 (50%) came from BIDMC and 1400 (50%) came from MGH. CHF prevalence in our cohort was 425 (15.9%) out of 1400 notes in the BIDMC dataset and 445 (15.2%) out of 1400 notes in the MGH dataset. Note length varied from 1907 to 60,870 characters.

**Table 2:** Cohort characteristics.

Characteristics		Site and group classification										
		Mass General Hospital						Beth Israel Deaconess Medical Center				
		*ICD*^a^+ /MED^b^+	*ICD*+ /MED–	*ICD*– /MED+	*ICD*– /MED–	Total	*ICD*+ /MED+		*ICD*+ /MED–	*ICD*+ /MED+	*ICD*+ /MED–	Total
Patients, n		347	348	348	350	1398	41		0	220	167	428
Notes, n		350	350	350	350	1400	350		—^c^	700	350	1400
Positive cases based on manual annotation, n (%)^d^		151 (43.1)	184 (52.6)	44 (12.6)	46 (13.1)	—	135 (38.6)		—	192 (27.4)	118 (33.7)	—
Age (years), mean (SD)		73.8 (12.3)	74.5 (13.1)	65.4 (15.2)	57.3 (23.4)	67.7 (18.0)	71.2 (11.9)		—	63.8 (13.1)	65.0 (14.1)	65.0 (13.8)
**Sex, n (%)^e^**												
	Female	175 (50.4)	178 (51.1)	196 (56.3)	214 (61.1)	763 (54.8)	20 (48.8)		—	110 (50)	85 (50.9)	215 (50.2)
	Male	172 (49.6)	170 (48.9)	152 (43.7)	136 (38.9)	630 (45.2)	21 (51.2)		—	110 (50)	82 (49.1)	213 (49.8)
**Race and ethnicity, n (%)^d^**												
	Black or African American	22 (6.3)	15 (4.3)	17 (4.9)	15 (4.3)	69 (4.9)	0 (0)		—	0 (0)	0 (0)	0 (0)
	Hispanic	14 (4.0)	22 (6.3)	25 (7.2)	19 (5.4)	80 (5.7)	2 (4.9)		—	20 (9.1)	13 (7.8)	35 (8.2)
	Asian	7 (2)	4 (1.1)	4 (1.1)	7 (2)	22 (1.6)	0 (0)		—	8 (3.6)	5 (3)	13 (3)
	White	295 (85)	305 (87.6)	289 (83)	296 (84.6)	1185 (85.1)	29 (70.7)		—	141 (64.1)	116 (69.5)	286 (66.8)

^a^ICD: International Classification of Diseases.

^b^MED: medications.

^c^Not applicable.

^d^Percentages are based on the number of notes as the denominators.

^e^Percentages are based on the number of patients as the denominators.

### Model Performance

**Figure 2:**
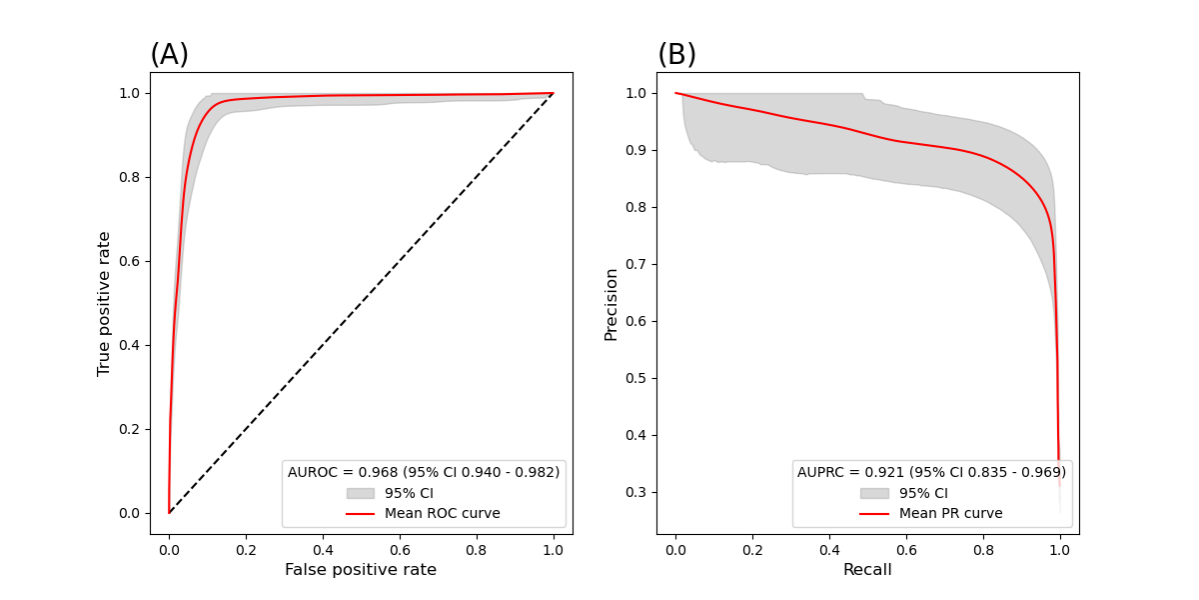
Model performance using the combined MGH and BIDMC test data. (A) Receiver operating characteristic (ROC) curve using logistic regression with notes, ICD codes, and medications as input. The area under the ROC curve (AUROC) was 0.968 (95% CI 0.940-0.982). The optimal cutoff (according to the Youden index) achieved a false positive rate of 11.9% (230/1903; ie, specificity at 88.1%) and a true positive rate of 97.7% (852/870; ie, sensitivity at 97.7%). (B) Precision-recall (PR) curve using logistic regression with notes, ICD codes, and medications as input. The area under the PR curve (AUPRC) was 0.921 (95% CI 0.835-0.969). The optimal cutoff achieved a recall of 96.78% (842/870; ie, sensitivity at 96.78%) and a precision of 80.88% (842/1081). BIDMC: Beth Israel Deaconess Medical Center; ICD: International Classification of Diseases; MGH: Mass General Hospital.

Table 3 shows performances from all three models and four input modalities (9 models in total). Models that used *ICD* codes, medications, and notes from both hospitals combined performed the best, followed by models that used notes only. Models that used *ICD* codes or medications only performed substantially worse. Meanwhile, logistic regression models performed the best, followed by random forests models, and then RoBERTa models. The receiver operating characteristic and precision-recall curves for the best-performing model and input modalities are shown in Figure 2.

**Table 3:** AUROC^a^ and AUPRC^b^ on the combined MGH^c^ and BIDMC^d^ test data for the logistic regression, random forests, and RoBERTa embeddings models, using different types of model input (ICD^e^ codes, medications, and notes). The best performances are italicized.

Input	Logistic regression			Random forests			RoBERTa	
	AUROC (95% CI)	AUPRC (95% CI)	AUROC (95% CI)		AUPRC (95% CI)	AUROC (95% CI)		AUPRC (95% CI)
*ICD* codes, medications, and notes	*0.968 (0.940-0.982)*	*0.921 (0.835-0.969)*	0.962 (0.719-0.991)		0.894 (0.720-0.985)	0.866 (0.821-0.904)		0.766 (0.632-0.853)
Notes only	0.964 (0.942-0.975)	0.901 (0.805-0.956)	0.963 (0.916-0.976)		0.899 (0.659-0.968)	—^f^		—
*ICD* codes only	0.646 (0.580-0.826)	0.557 (0.486-0.716)	0.646 (0.595-0.835)		0.557 (0.492-0.720)	—		—
Medications only	0.639 (0.338-0.875)	0.428 (0.235-0.651)	0.644 (0.326-0.936)		0.459 (0.320-0.779)	—		—

^a^AUROC: area under the receiver operating characteristic curve.

^b^AUPRC: area under the precision-recall curve.

^c^MGH: Mass General Hospital.

^d^BIDMC: Beth Israel Deaconess Medical Center.

^e^ICD: International Classification of Diseases.

^f^Not applicable.

### External Validity

**Figure 3:**
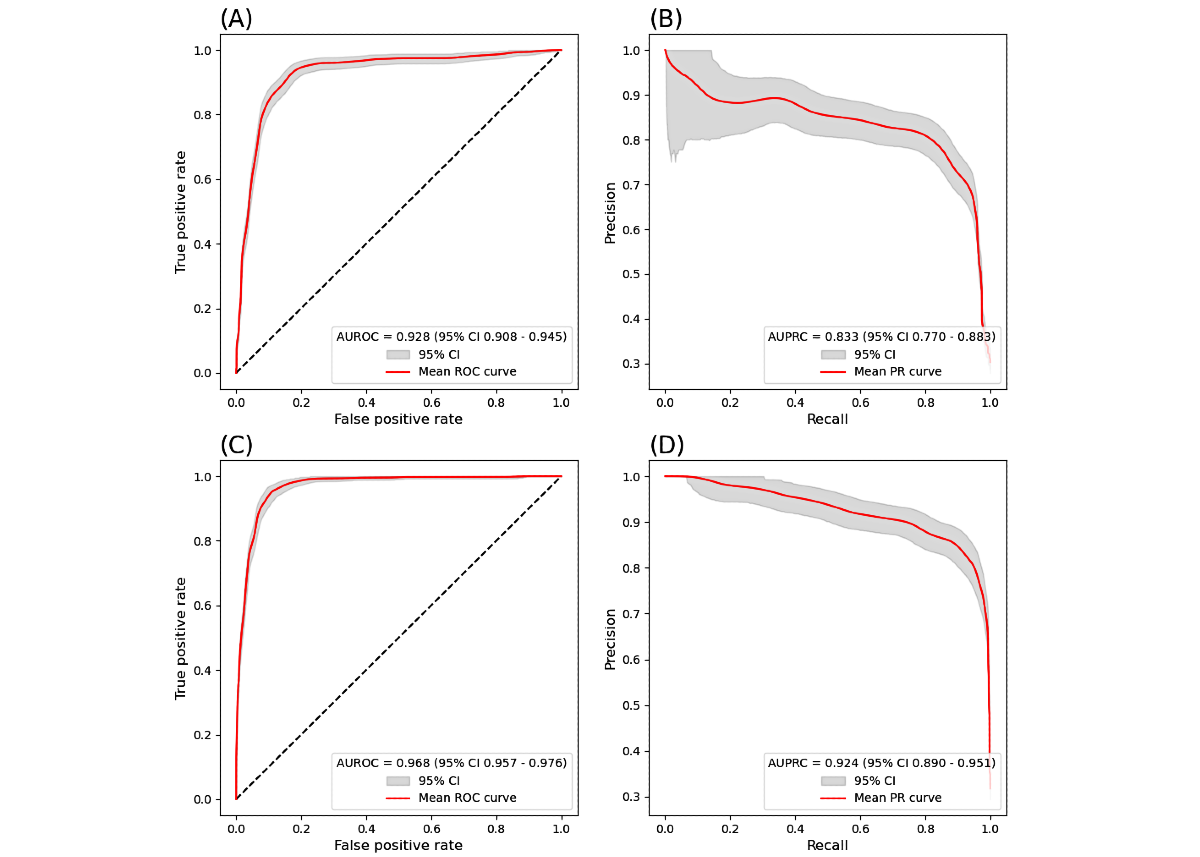
Model performance of training exclusively on one hospital’s data and testing on the other’s data. (A) and (B) Performance of training on MGH data and testing on BIDMC data. (C) and (D) Performance of training on BIDMC data and testing on MGH data. AUPRC: area under the precision-recall curve; AUROC: area under the receiver operating characteristic curve; BIDMC: Beth Israel Deaconess Medical Center; MGH: Mass General Hospital; PR: precision-recall; ROC: receiver operating characteristic.

To investigate how the model generalizes, we also obtained the performance when training on data from one site and testing on data from the other site (Figure 3 and Table 4). Training on BIDMC data and testing on MGH data showed significantly higher performance than training on MGH data and testing on BIDMC data. The performance of training on BIDMC data and testing on MGH data was similar to the cross-validated performance shown in Figure 2.

The results in Figure 3 show high external validity of the model due to its ability to perform well with training on one hospital dataset and subsequent testing on the other hospital dataset.

**Table 4:** Logistic regression coefficients from using the model trained with notes, ICD^a^ codes, and medications. Unexpected results are discussed in the Error Analysis section. Small coefficients for some input variables were expected and removed at the time of model deployment.

Features	Logistic regression coefficients
CHF^b^	2.39
Congestive heart failure	1.89
With CHF	1.34
CHF father	–1.15
Histori congestive heart failure	0.94
CHF mother	–0.80
No CHF	–0.72
I50	0.68
Dx^c^ CHF	0.64
Exacerb CHF	0.61
With congestive heart failure	–0.60
[] CHF	–0.54
Ascites	0.54
() Congestive heart failure	0.52
Diagnosis congestive heart failure	0.49

^a^ICD: International Classification of Diseases.

^b^CHF: congestive heart failure.

^c^Dx: diagnosis.

### Feature Importance

Feature importance for the best-performing model (logistic regression) is shown in Table 4. The top-3 positive predictors were the words and phrases “CHF,” “congestive heart failure,” and “with CHF.” The top-3 negative predictors were “CHF father,” “CHF mother,” and “no CHF.”

In Table 4, the negative weights of “CHF father” and “CHF mother” came from clinicians’ discussion of family history in the note. Brackets such as those in “[] CHF” or “() CHF” were purposely left in each note as they were often part of a medical questionnaire to obtain the medical history for new patients. Coefficients of “with CHF” and “with congestive heart failure” were given negative and positive weights, respectively, possibly due to the high collinearity between them.

### Error Analysis

We investigated reasons for misclassification in cases where the model output disagreed with manual labels. For false positives, reasons included patients with symptoms typical for CHF who had not been given a formal diagnosis of CHF, patients with a family medical history of CHF, cases where negation was expressed unusually (eg, “I do not feel like this is consistent with congestive heart failure”), and notes that had templated preoperative instructions or medication descriptions (eg, “tell your doctor if you have CHF”). For false negatives, reasons included notes explaining a noncardiac medical visit but mentioning a history of CHF, and patients with a diagnosis of CHF no longer experiencing symptoms.

### Performance in Unselected or Random EHR Samples

The sample used for training and testing was, by construction, enriched for “positive” cases; that is, there were more “*ICD*+/MED+,” “*ICD*–/MED+,” and “*ICD*+/MED–“ cases and fewer “*ICD*–/MED–” cases than would be present in a random sample. To estimate the expected performance in a general hospital population, we randomly sampled an additional 500 patients from BIDMC and 500 patients from MGH. Using our final fitted model from logistic regression methods (explained in detail above), we classified these 1000 patients as per Table 5. Using the prevalence values (p+/+, p+/–, p–/+, and p–/–) along with the corresponding overall model error rate (P_e_+/+, P_e_+/–, P_e_–/+, and P_e_–/–), the estimated performance within the overall hospital population was calculated as follows:

P(E) = (P_e_+/+ × p+/+) + (P_e_+/– × p+/–) + (P_e_–/+ × p–/+) + (P_e_–/– × p–/–)

= (0.074 × 0.1) + (0.074 × 0.098) + (0.079 × 0.0088) + (0.094 × 0.0021)

= 0.016

= 1.6%

Thus, the overall error rate in the general hospital population was estimated to be 1.6%.

**Table 5:** The statistics used to derive the overall error rate in the general hospital population, stratified by the four groups.

Statistics	Group classification			
	*ICD*^a^+/MED^b^+	*ICD*+/MED–	*ICD*–/MED+	*ICD*–/MED–
Error rate in the original samples for model training and testing (P_e_), n/N (%)	52/700 (7.4)	26/350 (7.4)	83/1050 (7.9)	66/700 (9.4)
Prevalence in additional random samples (p), n/N (%)	14/140 (10)	4/41 (9.8)	3/342 (0.9)	1/477 (0.21)

^a^*ICD*: *International Classification of Diseases*.

^b^MED: medications.

## Discussion

### Principal Findings

In this work, we present a highly accurate machine learning model that uses NLP to identify patients diagnosed with CHF in EHRs. The simple modeling technique demonstrated excellent discrimination for the presence or absence of CHF. Testing across two hospitals demonstrated the generalization of our model. This model provides a strong foundation for automated, scalable phenotyping of CHF at singular hospitals with big data and enable large-scale, EHR-based, epidemiological outcomes research. This work also provides the groundwork for future clinical applications, with the potential for improving clinical decision-making and patient outcomes by providing classification for diseases overlooked by clinicians. Such models could be incorporated into the current clinical workflow by inputting structured and unstructured data from visit notes by providers and comparing inconsistencies between providers’ interpretation of patients’ problems and what the model predicts could be an issue. This would ensure greater patient care and better use of technological advancements. The best-performing model was also simplistic, ensuring greater interpretability, computational efficiency, scalability, and cost-effectiveness of implementation of these methods.

In a previous study, EHR-based phenotyping of CHF was performed by comparing a deep learning model’s predictions against *ICD* code–based diagnoses using a large cohort from a single hospital [10]. This work, however, did not address the common issues of *ICD* codes being an inaccurate representation of the true diagnosis and the generalizability of the model. Other studies have used inductive logic programming to create helpful rules for phenotyping patients with several diagnoses [8]. These studies, however, did not address how such approaches can be scaled to larger and noisier data as found in clinical databases.

Differences in generalizability between the two hospital cohorts need future research. When comparing training on a single hospital cohort and testing on the other, training on BIDMC data and subsequent testing on MGH data had a slight improvement compared to the reverse. This could be due to different hospitals or clinicians classifying CHF in different ways (such as heart failure with preserved ejection fraction, heart failure with reduced ejection fraction, etc), as well as inconsistencies of information provided in unstructured text (history of present illness, family history, etc) between hospitals.

The performance of the notes-only models was not significantly different from models using notes, *ICD* codes, and medications, suggesting future avenues of research comparing the necessity of obtaining such information for model improvement. Much of the information presented by medication lists and *ICD* codes is already found within clinical notes and could be redundant information.

Our work has important limitations. First, data used for training and testing were collected exclusively from two large hospitals in Boston, Massachusetts. Generalizability to other hospital and health care systems needs to be explored, understanding that these results are biased toward the Massachusetts population and use data from primarily top academic institutions. Second, echocardiography reports were not used. Future models that incorporate echocardiography records may have even better performance for CHF [17]. Third, the lack of racial and ethnic diversity in the saturated sample with CHF may inhibit generalizability.

### Conclusion

We developed and validated a highly accurate NLP model for identifying patients with CHF from EHRs. This provides a foundation for large-scale, automated phenotyping of patients with CHF, enabling more efficient and scalable epidemiologic and outcomes research compared to manual chart review.

## Abbreviations

AUPRC: area under the precision-recall curve
AUROC: area under the receiver operating characteristic curve
BIDMC: Beth Israel Deaconess Medical Center
CHF: congestive heart failure
EHR: electronic health record
ICD: International Classification of Diseases
MGH: Mass General Hospital
NLP: natural language processing

## Data Availability

The deidentified electronic health record dataset is available upon reasonable request from the corresponding author. The code and intermediate results are available on GitHub [16].
